# Drug utilization in geriatric psychiatric patients in the emergency department—a cohort study under real-world conditions

**DOI:** 10.1177/20451253251339373

**Published:** 2025-05-16

**Authors:** Martin Schulze Westhoff, Sophie Bannasch, Johannes Heck, Stefan Bleich, Sebastian Schröder, Adrian Groh

**Affiliations:** Department of Psychiatry, Social Psychiatry and Psychotherapy, Hannover Medical School, Carl-Neuberg-Str. 1, Hannover 30625, Germany; Department of Psychiatry, Social Psychiatry and Psychotherapy, Hannover Medical School, Hannover, Germany; Institute for Clinical Pharmacology, Hannover Medical School, Hannover, Germany; Department of Psychiatry, Social Psychiatry and Psychotherapy, Hannover Medical School, Hannover, Germany; Department of Psychiatry, Social Psychiatry and Psychotherapy, Hannover Medical School, Hannover, Germany; Department of Psychiatry, Social Psychiatry and Psychotherapy, Hannover Medical School, Hannover, Germany

**Keywords:** drug safety, emergency department, FORTA, geriatric psychiatry, potentially inappropriate medications, PRISCUS

## Abstract

**Background:**

Psychiatric emergencies include agitation, substance-related (e.g., withdrawal) symptoms, and suicidal as well as self-harming behavior and require interdisciplinary management. Drug treatment of geriatric patients in emergency situations may be complicated by adverse drug reactions (ADRs).

**Objectives:**

This study aimed to investigate prescriptions of potentially inappropriate medications (PIMs) and potential drug–drug interactions (DDIs) in the context of geriatric psychiatric emergencies in the emergency department (ED).

**Design:**

Retrospective single-center study.

**Methods:**

The medication lists of 87 consecutively acquired geriatric patient cases receiving pharmacological treatment between January 2018 and December 2022 in a psychiatric emergency department were analyzed. Herein, utilizing the PRISCUS 2.0 list and the Fit fOR The Aged (FORTA) classification, prescriptions of PIMs were assessed, and DDIs were classified with the aid of the drug interaction program AiDKlinik® (Arzneimittel-Informations-Dienste, Dosing GmbH, Heidelberg, Germany).

**Results:**

A total of 94 drugs were administered during treatment in the ED. The total number of drugs per patient was on average 5.9 1 (median: 5; interquartile range: 4) hereafter. 77.7% of the newly prescribed drugs were PIMs according to the PRISCUS 2.0 list, while 18.1% were designated as therapeutic alternatives to PIMs. 70.2% and 22.3% of the newly recommended drugs were FORTA category C and D drugs, respectively. An average of 0.8 (median: 0; interquartile range: 1) potential DDIs existed before psychiatric ED treatment, and 0.9 (median: 0; interquartile range: 2) potential DDIs thereafter (*p* = 0.002). Coercive measures—such as administration of medication against the patient’s will—were rarely required in the study population.

**Conclusion:**

The majority of all drug prescriptions for the treatment of geriatric psychiatric emergencies were categorized as PIMs according to the PRISCUS 2.0 list and the FORTA classification. However, it should be noted that these PIM classification systems were not specifically designed for geriatric psychiatric settings. The number of potential DDIs was significantly higher after drug administration in the ED than before, which should prompt the monitoring of certain clinical parameters in the further course of treatment.

## Introduction

Treating patients with psychiatric disorders in the emergency department (ED) is an enormous challenge for all involved professions.^1,2^ Psychiatric emergencies (PEs) can present in various forms and include suicidal or self-harming behavior, intoxication, and acute psychotic states.^1,2^ To manage PEs appropriately, suitable pharmacotherapeutic approaches are often required, accompanied by psychotherapeutic techniques.^1^ In addition, patients with psychiatric disorders often also suffer from acute somatic comorbidities requiring consultation with other medical disciplines.^3,4^ In certain cases, there is also the risk that acute somatic diseases may be misdiagnosed as psychiatric disorders (e.g., hyperparathyroidism).^3^ Increasing admission rates to EDs due to psychiatric symptoms illustrate the need for developing appropriate strategies to manage PEs.^5,6^

Geriatric psychiatric patients represent a special population in EDs.^7,8^ PEs in this patient group particularly include—among others—agitation, which is often a behavioral abnormality in neurocognitive disorders such as dementia.^8^ Moreover, with a prevalence of about 15% among geriatric patients in EDs, delirium as a multiform state of mental confusion has to be considered in this patient group.^9^

Diverse aspects complicate the pharmacological treatment of geriatric patients.^10^ Older persons are particularly susceptible to adverse drug reactions (ADRs) due to altered pharmacodynamic and pharmacokinetic properties and changes in organ function (e.g., increased permeability of the blood-brain barrier or reduced renal and liver functions).^10,11^ This is further complicated by multimorbidity often present in older age, which in turn can lead to polypharmacy.^10,11^ Geriatric patients are also often prescribed potentially inappropriate medications (PIMs), which bear an unfavorable benefit-to-risk ratio and further increase the risk of ADRs.^12,13^

Other previous studies have shown that the use of benzodiazepines is frequent in geriatric PEs.^14,15^ Use of this drug class increases the risk of falls or cognitive impairment in geriatric patients instead.^16^ Therefore, waiving psychotropic drugs in cases of delirium is frequently recommended, and reorientation measures should be the focus of treatment.^17,18^ The use of psychotropic drugs for managing geriatric PEs has so far been insufficiently investigated under real-world conditions, particularly in EDs.

To date, one comparable study by Jalgaonkar et al.^19^ with a prospective, observational design exists in the literature, which investigated prescriptions for the treatment of PEs. However, the Jalgaonkar et al.^19^ study was conducted in India and did not include geriatric patients; hence, an information gap about the treatment of PEs in geriatric patients in Germany exists.

To address this information gap, the present study investigated prescriptions of PIMs and potential drug–drug interactions (DDIs) in the context of geriatric PEs in the ED of a large university hospital in Germany. Medication lists of geriatric patients treated in the psychiatric ED, who were administered medication in the ED, served as the basis. DDIs before and after drug prescriptions in the ED were evaluated. Furthermore, frequency and characteristics of PIM prescriptions were studied based on the most commonly used PIM classification systems in Germany, the PRISCUS list 2.0 (priscus (Latin), ancient, venerable) and the Fit fOR The Aged (FORTA) classification.^20,21^

## Methods

### Ethics approval

This study was approved by the Ethics Committee of Hannover Medical School (No. 10767_BO_K_2023) and adheres to the Declaration of Helsinki (1964) and its later amendments (current version from 2024).

### Eligibility criteria

Patients were enrolled in the study (i) if they were ⩾65 years of age, (ii) if they were treated in the psychiatric ED of Hannover Medical School between January 2018 and December 2022, (iii) if they were administered psychotropic drugs during treatment in the ED, and (iv) if they or their legal representative had provided written informed consent that patient-related data be used for clinical research. Patients received treatment in the ED for a psychiatric condition. Hannover Medical School is a large university hospital and tertiary care referral center in northern Germany. During the length of the study, 12,854 patient cases were treated in the psychiatric ED, 1783 of which referred to individuals aged ⩾65 years. There were no specific exclusion criteria. Psychiatric patients are treated in an interdisciplinary emergency department and, if inpatient treatment is indicated, they are transferred to a geriatric psychiatry ward for further treatment as soon as possible. Psychotropic drugs refer to drugs that affect brain function and are used to treat psychiatric and neurological disorders. These include antidepressants, antipsychotics, anxiolytics, and mood stabilizers, targeting conditions such as depression, schizophrenia, anxiety, and bipolar disorder.

The reporting of this study conforms to the STROBE statement (Supplemental Material).^22^

### Medication chart reviews, PIM classification systems, drug interaction checks, and demographic characteristics

Medication charts of enrolled patients were analyzed before and after psychiatric ED treatment by an expert panel of specialists in psychiatry and clinical pharmacology. Newly prescribed drugs were recorded for each patient and were assessed with the aid of the PRISCUS 2.0 list and the FORTA classification.

Published in 2010, the PRISCUS list was the first PIM classification explicitly tailored to the German pharmaceutical market. It contains recommendations on the use of 83 drugs classified as PIMs and lists possible therapeutic alternatives to PIMs.^23^ A more comprehensive and revised version was published in 2023 as PRISCUS 2.0 list and tabulates 187 drugs as PIMs.^20^ This resulted in a more detailed categorization of individual drug classes, such as nonsteroidal anti-inflammatory drugs (NSAIDs) or antipsychotics. Within our study, newly prescribed drugs were classified as PIMs (according to the PRISCUS 2.0 list), non-PIMs (i.e., drugs not listed as PIMs in the PRISCUS 2.0 list), and suitable therapeutic alternatives to PIMs (according to the PRISCUS 2.0 list) by the expert panel described above. The classification of some drugs as PIMs or therapeutic alternatives is partly based on the prescription period and dosage. Therefore, certain prescriptions (e.g., pipamperone or risperidone) were only classified as PIMs if they were prescribed as long-term medication after administration in the ED.

The FORTA classification was validated for the first time in 2014 and applies to people aged ⩾65 years and is explicitly tailored to the German pharmaceutical market.^24^ This classification system is an implicit instrument that can only be used if medical information about the patient is available, too. Currently, in its fourth iteration, the FORTA classification divides a total of 299 different drugs into categories A–D depending on the therapeutic indication: A = indispensable drugs in the pharmacological treatment of older people; B = drugs with proven or obvious efficacy in older people; C = drugs with questionable efficacy–safety profiles in older people; D = drugs that should be avoided in older people.^21^ In this study, drugs not mentioned in the FORTA classification were classified as “Not labeled.”

Drug interaction checks were performed for all medication lists before and after administration of psychotropic drugs in the ED with the tool “Medibox” of AiDKlinik^®^ (Arzneimittel-Informations-Dienste, Dosing GmbH, Heidelberg, Germany). Only potential DDIs that were classified as “moderate,” “severe” or “contraindicated combination” by AiDKlinik^®^ were considered for statistical analysis.

Demographic characteristics—that is, age, sex, and International Statistical Classification of Diseases and Related Health Problems 10th Revision (ICD-10) diagnoses—were retrieved from the patient records. Records also provided information on whether patients received coercive measures (forced medication and restraints) and whether they were involuntarily admitted.

### Statistical analysis

**Figure 1.**
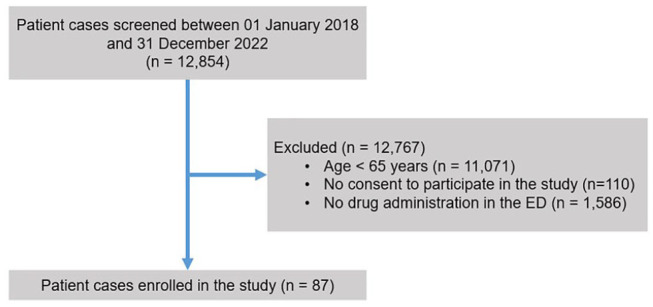
Flow of patients. Patients of the psychiatric ED could be enrolled in the study if they were administered medication during ED treatment. Furthermore, they had to be ⩾65 years of age and they or their legal guardian had to provide written informed consent. ED, Emergency Department.

Quantitative variables are depicted as means ± standard deviations and also as medians with interquartile ranges (IQRs). For categorical variables, absolute and relative frequencies were calculated. All statistical analyses were performed with IBM^®^ SPSS^®^ Statistics for Windows, version 28 (Armonk, New York, NY, USA). *p* Values < 0.05 were considered statistically significant. Testing for normal distribution was carried out by utilizing the Shapiro–Wilk test. For comparison of the number of potential DDIs before and after drug treatment in the ED, Wilcoxon signed rank tests were applied (Figure 1).

## Results

### Study population and drug recommendations

Of 12,854 screened patient cases, 87 cases involving 79 individual patients met the eligibility criteria and were included in the study. The higher number of patient cases as compared to the number of individual patients is explained by returners. The main reason for exclusion was age <65 years. The mean age of the study population (*n* = 87) was 74.1 ± 7.4 (median: 74; interquartile range: 11) years, and 62.1% (54/87) of the patients were female (Table 1). Substance use disorder was the most frequent psychiatric diagnosis in the study population (43.7%; 38/87), followed by dementia (28.7%; 25/87) and depression (23.0%; 20/87). Other psychiatric disorders, including personality disorders and anxiety and panic disorders, were also common in the study population (29.9%; 26/87). The most prevalent somatic comorbidity was arterial hypertension (35.6%; 31/87). In the majority of cases, patients were admitted voluntarily to a hospital (88.5%; 77/87), while involuntary admissions were less frequent (11.5%; 10/87).

**Table 1.** Characteristics of the study population (*n* = 87). The mean age ± standard deviation of the study population was 74.1 ± 7.4 years.

Variables	*n*	%
Sex		
Female	54	62.1
Male	33	37.9
Psychiatric diagnoses^a^		
Depression^b^	20	23.0
Schizophrenia or schizophreniform disorder^c^	12	13.8
Mental and behavioral disorders due to use of alcohol, tobacco, sedatives, or hypnotics^d^	38	43.7
Dementia^e^	25	28.7
Delirium^f^	16	18.4
Other psychiatric disorder(s)	26	29.9
Somatic diagnoses^a^		
Arterial hypertension	31	35.6
Coronary heart disease	6	6.9
Chronic heart failure	3	3.4
Atrial fibrillation	9	10.3
Status post-stroke	2	2.3
Type-2 diabetes mellitus	9	10.3
Chronic obstructive pulmonary disease	7	8.0
Hypothyroidism	9	10.3
Urinary tract infection	3	3.4
Other somatic disorder(s)	69	79.3
Main reasons for drug prescription		
Agitation	37	42.5
Suicidality and self-harming behavior	6	6.9
Substance-related (e.g., withdrawal symptoms)	29	33.3
Acute psychosis	15	17.2
Treatment basis		
Voluntary admission	77	88.5
Involuntary admission	10	11.5

a Patients could have more than one diagnosis.

b ICD-10 F32, F33.

c ICD-10 F06.2, F2X.

d ICD-10 F10, F13, F17.

e ICD-10 F00, F01, F02, F03.

f ICD-10 F05.

ICD-10, International Statistical Classification of Diseases and Related Health Problems 10th Revision.

In total, 94 drugs were administered to patients during treatment in the ED, mainly due to agitation (42.5%; 37/87) and substance-related symptoms (33.3%; 29/87). Forced medication and restraints were applied only in one patient case in the study population.

Including given medication during ED treatment, patients received an average of 5.9 ± 4.1 (median: 5; interquartile range: 4) drugs. The most frequently administered drugs in the ED were lorazepam (34.2%; 34/94), pipamperone (25.5%; 24/94), and oxazepam (17.0%; 16/94).

### Potentially inappropriate medications for older people according to the PRISCUS 2.0 list

Of the 94 drugs administered during ED treatment, 77.7% (73/94) were classified as PIMs according to the PRISCUS 2.0 list (PRISCUS-PIMs), whereas only 18.1% (17/94) were identified as suitable therapeutic alternatives to PIMs based on the same classification. The most frequently administered PRISCUS-PIMs were lorazepam (46.6%; 34/73), oxazepam (21.9%; 16/73), and long-term (i.e., >6 weeks) pipamperone (16.4%; 12/73) (Table 2). On the other hand, pipamperone (70.6%; 12/17) and melperone (11.8%; 2/17) were the most frequently administered drugs listed as suitable therapeutic alternatives to PIMs when only applied for short periods (i.e., ⩽6 weeks).

**Table 2.** Absolute and relative frequencies of potentially inappropriate medications for older people (i.e., ⩾65 years of age) according to the PRISCUS list 2.0 that were newly prescribed in the study population.

Potentially inappropriate medication	*n*	%
All potentially inappropriate medications	**73**	**100**
Lorazepam	34	46.6
Oxazepam	16	21.9
Pipamperone > 120 mg/day, >6 weeks	12	16.4
Haloperidol	5	6.8
Midazolam	2	2.7
Carbamazepine	1	1.4
Diazepam	1	1.4
Flupentixol	1	1.4
Risperidone > 6 weeks	1	1.4

Bold no significances to report.

PRISCUS-PIMs were applied in 82.8% (72/87) of all patient cases during ED treatment.

### Categorization of drug prescriptions according to the FORTA classification

Of the newly prescribed drugs, 0%, 2.1% (2/94), 70.2% (66/94), and 22.3% (21/94) were classified as FORTA categories A, B, C, and D, respectively. 5.3% (5/94) of the newly recommended drugs were not indexed in the FORTA classification.

In 74.7% (65/87) of all patient cases, at least one newly prescribed FORTA C drug was identified, while 24.1% (21/87) involved the new prescription of at least one FORTA D drug. The most frequently utilized FORTA C drugs were lorazepam (51.5%; 34/66) and pipamperone (36.4%; 24/66), whereas oxazepam (76.2%; 16/21) and haloperidol (23.8%; 5/21) represented the two FORTA D drugs applied in the study population (Table 3).

**Table 3.** Absolute and relative frequencies of FORTA class C drugs (i.e., drugs with questionable efficacy–safety profiles in older people) and FORTA class D drugs (i.e., drugs that should be avoided in older people) were newly administered in the study population during treatment in the ED.

Drug	*n*	%
FORTA class C drugs	**66**	**100**
Lorazepam	34	51.5
Pipamperone	24	36.4
Melperone	2	3.0
Midazolam	2	3.0
Risperidone	2	3.0
Carbamazepine	1	1.5
Diazepam	1	1.5
FORTA class D drugs	**21**	**100**
Oxazepam	16	76.2
Haloperidol	5	23.8

Bold no significances to report.

### Drug interaction checks

Drug interaction checks revealed that—on average—there existed 0.8 ± 1.2 (median: 0; interquartile range: 1) potential DDIs per patient before drug prescription in the ED and 0.9 ± 1.3 (median: 0; interquartile range: 2) DDIs afterward (*p* = 0.002). A total of 41.4% (36/87) of all patients displayed at least one drug-drug interaction (DDI) prior to drug administration, increasing to 43.7% (38/87) thereafter. Overall, there were 65 DDIs classified as “moderate” or “severe” combinations” in the study population before and 75 DDIs after drug prescription in the ED, while no contraindicated combinations were detected. Before drug prescription in the ED, 23.1% (15/65) of all DDIs were classified as “severe,” whereas all DDIs related to newly prescribed drugs in the ED were classified as “moderate.”

Before drug administration, the most frequent DDI categories were “disturbances of blood coagulation” (21.5%; 14/65), “hypotension” (16.9%; 11/65), and “electrolyte disturbances” (16.9%; 11/65).

Considering the given drugs in the ED, the most frequent DDI categories were “central nervous system (CNS) depressant effects” (24.0%; 18/75), “disturbances of blood coagulation” (18.7%; 14/75), and “hypotension” (16.0%; 12/75). The most frequent drug combinations with interaction potential related to drug utilization in the ED were the combinations of risperidone and pipamperone (40.0%; 4/10) and risperidone and melperone (30.0%; 3/10). Other newly administered drugs with interaction potential were lorazepam and propofol. In general, risperidone (24.0%; 18/75), pipamperone (12.0%; 9/75), and melperone (5.3%; 4/75) were also the psychotropic drugs most frequently involved in potential DDIs.

## Discussion

The present study examined aspects of medication safety during treatment in a psychiatric ED at a large German university hospital. A particular focus was placed on drug treatment in geriatric PEs in the ED. PIM prescriptions according to the PISCUS 2.0 list and the FORTA classification, as well as potential DDIs, were recorded before and after drug administration to assess safety and age adaptation of the prescription process. In this study, 87 patient cases were analyzed. The mean age was 74.1 years, with a majority being female (62.1%). Substance use disorder (43.7%) was the most common psychiatric diagnosis, followed by dementia (28.7%) and depression (23.0%). Importantly, 77.7% of administered drugs were classified as PIMs according to the PRISCUS 2.0 list, and 82.8% of all patient cases were affected by at least one PRISCUS-PIM. According to the FORTA classification, the majority of newly prescribed drugs were categorized as FORTA C (70.2%), while 22.3% were FORTA D, indicating a significant portion of prescriptions with potential safety concerns. Drug interaction checks revealed that the proportion of patients with at least one DDI increased from 41.4% before drug administration to 43.7% thereafter, with CNS depressant effects being the most common DDI category after ED treatment.

To date, only one comparable study by Jalgaonkar et al.^19^ with a prospective, observational design exists. This study investigated prescriptions for psychiatric emergencies but did not include geriatric patients.^19^ Among 110 participants, it was stated that around 4.2 drugs per patient were administered for emergency treatment.^19^ Prescription was most frequently due to intoxication with psychotropic substances or schizophrenic psychoses.^19^ Risperidone, lorazepam, and clonazepam were the most frequently used drugs in this study from India.^19^ In accordance, risperidone and lorazepam were also the most frequently used drugs in our study, alongside pipamperone. Substance use disorders were also the most frequently included diagnosis group, and substance-related symptoms were the second most common reason for drug administration. The composition of our study population was very similar in terms of age, gender, and somatic comorbidities to those in studies that also referred to geriatric psychiatric patient groups.^13,25^

The present study has shown that in geriatric PEs, only 18.1% of all prescriptions were classified as a therapeutic alternative according to the PRISCUS 2.0 list. So, the proportion of PIM prescriptions based on the PRISCUS 2.0 list is significantly higher than previously reported.^13,25^ However, comparability between these studies is difficult for various reasons. First, the results of our work relate to the treatment of PEs in the ED setting, whereas other studies were based on routine data from the treatment of outpatients and inpatients. Furthermore, the use of some drug classes classified as PIM, such as benzodiazepines or certain antipsychotics such as haloperidol, is often unavoidable in PEs due to a lack of alternatives, which is now also included as a discussion point in the PRISCUS 2.0 list.^20^ In addition, PIM prevalences of preliminary studies generally referred to the first version of the PRISCUS list, while the updated version from 2023 was used as the basis for our work. This version now includes recommendations for 177 drugs and is significantly more restrictive than the first version, which only included information on 83 drugs. In line with this, a recent study examining prescribing in geriatric inpatients based on the PRISCUS 2.0 list found that 77.1% of all patients received at least one PIM prescription.^26^

In the present study, most newly prescribed drugs were classified as FORTA C (70.2%) and FORTA D (22.3%), indicating an unfavorable risk-benefit ratio for a substantial proportion of prescriptions. Almost all patients in the study population received at least one PIM prescription according to the FORTA classification. One possible explanation for this high prevalence is that the FORTA classification, with recommendations for 299 drugs, is more extensive than the PRISCUS 2.0 list. Of note, neither the PRISCUS 2.0 list nor the FORTA classification is specifically tailored to geriatric psychiatric patients, and the benefit-risk ratio is rarely taken into account for psychiatric emergencies.

In contrast to the PRISCUS list, there are fewer studies reporting PIM prevalence based on the FORTA classification. A multicenter observational study by Krüger et al.^27^ found a significantly higher prevalence of PIM prescriptions among geriatric patients in Germany according to the FORTA classification compared to the PRISCUS list. The prevalence of PIM prescriptions according to the FORTA classification was recorded as 44.3% in another study focusing on a geriatric patient collective, whereas Schröder et al.^28,29^ reported a prevalence of 71.1% in geriatric patients with alcohol use disorder. These findings suggest that PIM prevalence according to the FORTA classification is consistently higher than in studies referring to the PRISCUS list.

The most frequently administered drugs listed as PIM according to the PRISCUS 2.0 list were lorazepam, oxazepam, and pipamperone. However, it should be noted that although pipamperone, which may be a therapeutic alternative to benzodiazepines in certain clinical scenarios, was categorized as a PIM when administered as a regular medication for a period of more than 6 weeks, its short-term use for the treatment of PEs was appropriate. According to the FORTA classification, lorazepam and pipamperone with label C and oxazepam and haloperidol with label D were the most frequently used PIMs. A study on prescribing behavior in geriatric patients in a German practice network also identified antipsychotic and antidepressant drugs as the most frequently used PIMs.^30^ In a study by Hefner et al.,^31^ lorazepam and haloperidol were also the most frequently prescribed PIMs in a geriatric psychiatric setting, alongside zopiclone and diazepam.

As agitation and substance-related symptoms were the most common reasons for drug administration in the present study, the use of lorazepam and oxazepam appears to be unavoidable. Although the delirogenic potential, increased risk of falls, and dependency potential of lorazepam and oxazepam must be considered, the use of these drugs appears to be more suitable than the use of long-acting benzodiazepines such as diazepam with its risk of cumulation.^16,32^ Available alternatives for the treatment of agitation or suicidality, such as low-potency antipsychotics like levomepromazine or chlorpromazine, also bear anticholinergic effects and the risk of extrapyramidal motor ADRs.^33^ The use of haloperidol for managing agitation, particularly in geriatric patients with agitation caused by intoxication, is the most well-researched option and currently lacks a compelling alternative.^33^

To evaluate medication safety in the context of geriatric PE treatment, potential DDIs were also recorded before and after drug administration in the ED in the present study. Preliminary studies have also evaluated potential DDIs as medication-related problems.^34,35^ A cross-sectional study from the USA reported that of 3055 community-dwelling older adults, almost a quarter had at least one potential DDI in their medication.^34^ An analysis of inpatients with dementia found that 76% showed at least one potential DDI.^35^ In that study, psychotropic drugs most frequently involved in DDIs were sertraline and citalopram, which, in combination with other drugs, led to an increased risk of bleeding.^35^ Our study found that the number of DDIs per patient was significantly higher when considering drugs administered in the ED than before treatment. The majority of additional DDIs were due to an interaction of risperidone with low-potency antipsychotics (melperone and pipamperone), with the risk of CNS depressant effects. At the same time, when combining risperidone with pipamperone or melperone, the QTc prolonging potential of all drugs involved has to be considered.^36^ Thus, the results of our study suggest that specific monitoring of vigilance, motor functions, and electrocardiogram (ECG) should be routinely conducted after drug administration for the treatment of geriatric PEs.

The recording and avoidance of coercive measures during psychiatric treatment is pivotal. Several studies have already attempted to identify possible risk factors for coercive measures.^37,38^ A retrospective analysis by Cole et al.^39^ of inpatient data from a German psychiatric hospital attributed male gender, aggression, and acute intoxication states with a higher risk of coercive measures. With regard to the geriatric psychiatric setting, a further retrospective study from Switzerland showed that 16.4% of all included patients had to undergo coercive measures during treatment.^40^ In that study, a higher risk of coercive measures was attributed to male patients with cognitive disorders and aggressive behavior.^40^ The results of our study documented that forced medication when treating geriatric PEs was necessary only rarely. Only in one of the 87 patient cases, an acute psychotic state to be treated by restraint and forced intramuscular drug administration. In the other cases in which hospitalization was involuntary, drug administration in the ED was performed with the patient’s consent.

### Limitations

The significance of our results is limited by the retrospective single-center study design. In addition, a large number of the initially screened potential study participants had to be excluded, as most of them were not administered medication in the ED and were younger than 65 years. Furthermore, the specialized setting of a university hospital cannot be directly extrapolated to peripheral psychiatric hospitals. The relatively small sample size also limits the generalizability of our findings. Finally, no systematic recording of ADRs took place. This, in turn, could serve as a starting point for further studies prospectively recording prescribing behavior in the treatment of geriatric PEs while also monitoring possible ADRs.

## Conclusion

In summary, the majority of drug prescriptions for the treatment of PEs in the ED were classified as PIMs according to the PRISCUS 2.0 list and the FORTA classification. PIM prevalence was significantly higher than in preliminary studies, which was due to the high rate of prescriptions for benzodiazepines and low-potency antipsychotics. Nevertheless, their use in the treatment of geriatric PEs is often unavoidable and generally appears beneficial. However, their long-term use as a regular medication should be avoided whenever possible. Instead, we call for a PIM classification specifically tailored to the treatment of geriatric PEs that should be devised in the future. In addition, a relevant number of potential DDIs were found to be associated with drug administration in the ED. For prevention and early detection of ADRs possibly caused by DDIs, comprehensive monitoring of various clinical parameters should be carried out following treatment in the ED.

## Supplemental Material

sj-docx-1-tpp-10.1177_20451253251339373 – Supplemental material for Drug utilization in geriatric psychiatric patients in the emergency department—a cohort study under real-world conditions Supplemental material, sj-docx-1-tpp-10.1177_20451253251339373 for Drug utilization in geriatric psychiatric patients in the emergency department—a cohort study under real-world conditions by Martin Schulze Westhoff, Sophie Bannasch, Johannes Heck, Stefan Bleich, Sebastian Schröder and Adrian Groh in Therapeutic Advances in Psychopharmacology

## Appendix

### Abbreviations

ADR adverse drug reaction

AiD Arzneimittel-Informations-Dienste (Drug Information Services)

CNS central nervous system

DDI drug–drug interaction

ECG electrocardiogram

ED emergency department

FORTA Fit fOR The Aged

ICD-10 International Statistical Classification of Diseases and Related Health Problems 10th Revision

PE psychiatric emergency

PIM potentially inappropriate medication (for older people)

PRISCUS Latin for ancient, venerable

## Declarations

**Ethics approval and consent to participate:** This study was approved by the Ethics Committee of Hannover Medical School (No. 10767_BO_K_2023) and adheres to the Declaration of Helsinki (1964) and its later amendments (current version from 2024). Patients were only included if they or their legal representative gave written consent for patient-related data to be used for clinical epidemiological research.

**Consent for publication:** Patients were only included if they or their legal representative gave written consent for patient-related data to be used for clinical epidemiological research.

**Availability of data and materials:** The data that support the findings of this study are available upon reasonable request from the corresponding author.
